# The Possible Influence of Non-synonymous Point Mutations within the FimA Adhesin of Non-typhoidal *Salmonella* (NTS) Isolates in the Process of Host Adaptation

**DOI:** 10.3389/fmicb.2017.02030

**Published:** 2017-10-17

**Authors:** Sahar Alshalchi, Shivdeep S. Hayer, Ran An, Jeannette Munoz-Aguayo, Christian Flores-Figueroa, Ryan Nguyen, Dale Lauer, Karen Olsen, Julio Alvarez, David Boxrud, Carol Cardona, Sinisa Vidovic

**Affiliations:** ^1^Department of Veterinary and Biomedical Sciences, University of Minnesota, Minnesota, MN, United States; ^2^Department of Population Medicine, University of Minnesota, Minnesota, MN, United States; ^3^Mid-Central Research and Outreach Center, University of Minnesota, Minnesota, MN, United States; ^4^Minnesota Poultry Testing Laboratory, University of Minnesota, Minnesota, MN, United States; ^5^Veterinary Diagnostic Laboratory, University of Minnesota, Minnesota, MN, United States; ^6^Public Health Laboratory, Minnesota Department of Health, Minnesota, MN, United States

**Keywords:** non-typhoidal *Salmonella*, host adaptation, adhesin FimA, receptor IroN, plasmid-encoded major fimbrial subunit PefA, salmonellosis

## Abstract

Non-typhoidal *Salmonella* (NTS) remains a global pathogen that affects a wide range of animal species. We analyzed a large number of NTS isolates of different host origins, including *Salmonella* Heidelberg (*n* = 80, avian), *S*. Dublin (50, bovine), *S*. Typhimurium var 5- (*n* = 40, porcine), *S*. 4,5,12,:i:- (*n* = 40, porcine), *S*. Cerro (*n* = 16, bovine), and *S*. Montevideo (*n* = 14, bovine), using virulence profiling of the *bcfC, mgtC, ssaC, invE, pefA, stn, sopB*, and *siiE* virulence-associated genes, a biofilm production assay, pulsed field gel electrophoresis, and the full-length sequencing of the *fimA* (adhesin) and *iroN* (receptor) genes. We determined a key amino acid substitution, A169 (i.e., threonine changed to alanine at position 169), in the FimA protein that changed ligand affinity of FimA toward N-acetyl-D-glucosamine. This finding clearly indicates the important role of non-synonymous single nucleotide polymorphism (nsSNPs) in adhesin functionality that may impact the host tropism of NTS. This nsSNP was found in *S*. Heidelberg and *S*. Cerro isolates. Although this was not the case for the IroN receptor, the phylogeny of this receptor and different host origins of NTS isolates were positively correlated, suggesting existence of specific host immune selective pressures on this unique receptor in *S. enterica*. We found that *pefA*, a gene encoding major fimbrial subunit, was the most-segregative virulence factor. It was associated with *S*. Heidelberg, *S*. Typhimurium var 5- and *S*. 4,5,12,:i:- but not with the rest of NTS strains. Further, we observed a significantly higher frequency of non-biofilm producers among NTS strains that do not carry *pefA* (42.5%) compared to *S*. Heidelberg (2.5%) and *S*. Typhimurium var 5- (7.5%) and *S*. 4,5,12,:i:- (0%). This study provides new insights into the host adaptation of avian and mammalian NTS isolates that are based on the bacterial antigens FimA and IroN as well as the interrelationships between host adaptation, overall genetic relatedness, and virulence potential in these NTS isolates.

## Introduction

Non-typhoidal *Salmonella* (NTS) remains a serious zoonotic pathogen worldwide (Bangtrakulnonth et al., 2004). The public health importance of NTS is underscored by the fact that from 2000 to 2008 infections caused by this pathogen accounted for ~1.2 million illnesses, with 23,000 hospitalizations and 450 deaths each year in the US alone (Scallan et al., 2011). The global situation is even worse: in excess of 1.3 billion people experience salmonellosis annually, with nearly 3 million deaths (O'Reilly et al., 2012; Keestra-Gounder et al., 2015). This incredibly diverse species can infect a wide range of hosts, including humans, poultry, cattle, and other domesticated and wild animals. Interestingly, within the population of NTS, there are *Salmonella* lineages that exhibit the narrow-host range (i.e., host-specialists), whereas certain NTS lineages have the wide-host range (i.e., host-generalists). For instance, *Salmonella enterica* subs. *enterica* serovar Enteritidis is capable of invading numerous host species, ranging from mammals, birds to reptiles (Altekruse et al., 2006; Bosch et al., 2016; Feasey et al., 2016). In a sharp contrast to *S*. Enteritidis, *S*. Gallinarum, a genetically closely related lineage, is strictly restricted to galiforme birds. In contrast to host-range diversity of NTS lineages, typhoidal serovars (TS) of *S. enterica* have a predilection for specific hosts. For instance, *S. enterica* serovar Typhi, and Paratyphi (e.g., causative agents of the systematic typhoid fever) are strict human pathogens (Gal-Mor et al., 2014). Despite the fact that these two groups of *S. enterica* spp. I, NTS and TS, share >96% DNA sequence identity (McClelland et al., 2001), the molecular bases for their host specificity differ profoundly. The mechanisms by which NTS serovars cross host barriers remain elusive. Recently, Yue et al. (2015), examining patho-adaptation of *S. enterica* serovar Typhimurium in diverse hosts, reported that non-synonymous single-nucleotide polymorphisms (nsSNPs) in certain antigen genes play an important role in host adaptation of NTS. They found distinct host-specific nsSNP signatures within the *fimH* gene (i.e., gene encoding the type 1 fimbrial adhesion) that may determine NTS host tropism. Moreover, nsSNPs may play a role in the host adaptation process of a narrower host range pathogen, such as *Neisseria gonorrhoeae*. Vidovic et al. (unpublished data) found that host immunity, acting on antigenic gene such the *tbpB* gene—which encodes an outer-membrane lipoprotein responsible for gonococcal transferrin-iron acquisition—can generate a series of nsSNPs during a single outbreak of *N. gonorrhoeae*, resulting in genetic diversification and host adaptation of the outbreak isolate. Gene gain or loss, also play a role in host adaptation of zoonotic and human pathogens. It has been found that invasive strains of *S*. Typhimurium and Enteritidis undergo a genome degradation to adapt to the new extraintestinal lifestyle. The genome degradation occurs via the formation of pseudogenes and the shedding of genes involved in the gut colonization and anaerobic catabolism of inflammation-derived nutrients (Feasey et al., 2016).

The aim of the present study was to investigate the role of nsSNPs in two antigen genes, *fimA*, and *iroN*, as well as the role of eight virulence genes, *bcfC, mgtC, ssaC, invE, pefA, stn, sopB*, and *siiE*, in the process of the host adaptation of NTS isolates obtained from avian (*S*. Heidelberg), bovine (*S*. Dublin, *S*. Cerro, *S*. Montevideo), and porcine (*S*. Typhimurium var 5-, *S*. 4,5, 4,5,12,:i:-) hosts. The entire collection of NTS isolates was analyzed for the presence of three virulence genes, *bcfC, pefA*, and *siiE*, involved in NTS colonization/biofilm formation and five virulence genes, *ssaC, invE, stn, sopB*, and *mgtC*, involved in invasion of the host. In addition to the screening for the three genes implicated in colonization/biofilm formation, we tested the all NTS isolates for their ability to form a biofilm, a crucial virulence phenotype that leads to chronic carriage and shedding of NTS (Hurley et al., 2014). Furthermore, we analyzed the role of nsSNPs in the host adaptation of NTS serovars using the full-length gene sequencing of *fimA* (i.e., gene encoding fimbriae that enable bacteria to colonize the epithelium of specific host organs) and *iroN* (i.e., gene that encodes outer membrane receptor of iron salmochelin). Both of these antigenic genes, *fimA* and *iroN*, have a high potential to influence the host adaptation of NTS. The adhesion FimA is important for attachment to enterocytes and promotes intestinal colonization of the host (Althouse et al., 2003). The IroN, a unique receptor of *S. enterica*, promotes a growth advantage to NTS over other gut micro biota, as this receptor uptakes metabolites excreted from other bacteria (Baumler et al., 1998). Our findings reveal interrelationships between host adaptation, overall NTS genetic relatedness, and NTS virulence.

## Materials and methods

### Collection of non-typhoidal *Salmonella* (NTS) isolates

The University of Minnesota Veterinary Diagnostic Laboratory (VDL), which is fully accredited by the American Association of Veterinary Laboratory Diagnosticians, serves as the veterinary microbiological reference center for the state of Minnesota. The Minnesota Poultry Testing Laboratory (MPTL), located in Willmar, MN serves as the authorized laboratory for the National Poultry Improvement Plan in Minnesota. During 2015, the VDL identified NTS isolates in 2049 clinical samples of avian (*n* = 1,406), porcine (*n* = 516), and bovine (*n* = 127) origin. For the present study, we selected NTS serovars most commonly associated with each of these three animal hosts according to VDL records (Table 1). In total, the collection had 240 NTS isolates, including 80 clinical isolates of bovine origin [i.e., *S. enterica* serovar Dublin (*n* = 50); *S. enterica* serovar Cerro (*n* = 16) and *S. enterica* serovar Montevideo (*n* = 14)], 80 clinical isolates of porcine origin [i.e., *S. enterica* serovar Typhimurium var 5- (*n* = 40) and *S. enterica* serovar 4,5,12:i:– (*n* = 40)] and 80 isolates associated with the poultry barns [i.e., *S. enterica* serovar Heidelberg (*n* = 80)]. Isolates of the bovine and porcine origins were received from the VDL, and isolates of the avian origin were received from the MPTL. Primary identification was undertaken at the National Reference laboratory, Ames, Iowa, using standard microbiological and serological methods. Isolates were stored at −80°C in Luria-Bertani (LB) broth (Difco) containing 10% glycerol. For each experiment in this study, fresh cultures derived from the frozen stocks were used.

**Table 1:** The most common serovars of NTS, isolated from the clinical samples of avian, bovine and porcine origins during 2015 at the Veterinary Diagnostic Laboratory, University of Minnesota.

**Name of serovars**	**Host origin of NTS isolates**			**Total (*n*)**
	**Avian (*n*)**	**Bovine (*n*)**	**Porcine (*n*)**	
*Salmonella* Heidelberg	389	6	22	417
*Salmonella* Typhimurium var 5-		2	77	79
*Salmonella* 4,5,12:i:-	78	5	75	158
*Salmonella* Dublin		51		51
*Salmonella* Cerro		17	3	20
*Salmonella* Montevideo	1	16	2	19
*Salmonella* Kentucky	197			197
*Salmonella* Uganda	181		4	185
*Salmonella* Reading	133			133
*Salmonella* Hadar	133			133
*Salmonella* Senftenberg	42		13	55
*Salmonella* Enteritidis	55			55
*Salmonella* Agona	1	3	47	51
*Salmonella* Mueenchen	46		4	50
*Salmonella* 4, 12:i:-		1	49	50
*Salmonella* St. Paul	45		1	46
*Salmonella* Derby			42	42
*Salmonella* Anatum	31		9	40
*Salmonella* Typhimurium	11	1	20	32
*Salmonella* Schwarzengrund	18	6	5	29

### Extraction of DNA

Non-typhoidal *Salmonella* (NTS) isolates were plated from frozen stocks on LB agar plates (Difco), followed by an overnight incubation at 37°C. Growth from an overnight culture was collected by a sterile loop and resuspended in 1 mL of 0.9% saline. After centrifugation at 10,000 X g for 1 min. the supernatant was removed and genomic DNA was extracted using the Qiagen DNeasy tissue kit (Qiagen Inc., Valencia, CA), according to the manufacturer's instructions.

### Virulence gene profiling

The NTS isolates were screened by PCR for the presence of eight virulence-associated genes, including three genes involved in NTS colonization/biofilm formation: fimbrial usher (*bcfC*), plasmid-encoded major fimbrial subunit (*pefA*), and non-fimbrial adhesion (*siiE*) as well as five genes involved in invasion of the host, invasion protein InvE (*invE*), secretion system apparatus outer membrane protein SsaC (*ssaC*), magnesium transport protein MgtC (*mgtC*), enterotoxin (*stn*), and inositol phosphate phosphatase SopB (*sopB*). Three multiplex PCR reactions were used to detect: (1) *mgtC*/*bcfC*; (2) *pefA*/*siiE*; and (3) *stn*/*sopB*. Identification of remaining two virulence-associated genes, *ssaC* and *invE*, was performed individually by employing conventional PCR methods. All primer sequences used for the virulence-profiling assay were designed in this study. *S. enterica* serovar Enteritidis ATCC 4931 was used as a positive control and *Escherichia coli* O157 strain B-1 (Vidovic and Korber, 2006) was used as a negative control. PCR amplification was carried out in 50 μL using a T100™ thermal cycler (Bio-Rad, Hercules, CA). Primers for the PCR assays used in this study are presented in Table 2.

**Table 2:** Primers used for detection of eight virulence-associated genes in the population of NTS isolates.

**Gene name**	**Protein function**	**Primer pair**	**Sequence of primers**	**Amplicon size (bp)**
*bcfC*	Fimbrial usher	*bcfC*-F	CCAGTACGCTGGCGGATAAT	177
		*bcfC*-R	TGTCATCGTCATAGCCGCTC	
*mgtC*	Magnesium transport protein MgtC	*mgtC*-F	ATTGGCGCGGAAAGACAATG	458
		*mgtC*-R	ATCGCGGCCTCTTTTACGAT	
*ssaC*	Secretion system apparatus outer membrane protein SsaC	*ssaC*-F	ACCTGGTTTGATGGCAGCAT	680
		*ssaC*-R	CCACTAGCACCACCGTCATT	
*invE*	Invasion protein InvE	*invE*-F	TCCAGTCGACGGACGAAATG	948
		*invE*-R	TAGTACGACGCTGTTCTGCC	
*pefA*	Plasmid-encoded major fimbrial subunit	*pefA*-F	CAGGGTTGTGCAAATCTGGC	165
		*pefA*-R	GCTGGCGTTAGCGTTTACAG	
*stn*	Enterotoxin	*stn*-F	CCGCGCCTTTACCCTCAATA	361
		*stn*-R	CAGGATGCCCAAAGCAGAGA	
*sopB*	Inositol phosphate phosphatase SopB	*sopB*-F	TTGTGGATGTCCACGGTGAG	644
		*sopB*-R	TTATAGGGTTCGCCGCCATC	
*siiE*	Non-fimbrial adhesion	*siiE*-F	AGAATCGCCTCGCTTACTCG	910
		*siiE*-R	ACGCACATCTTCCCAACGAT	

### Pulsed-field gel electrophoresis (PFGE)

All NTS isolates were characterized by the PFGE typing method, as previously described by the Centers for Disease Control and Prevention (CDC) PulseNet program (Ribot et al., 2006; Centers for Disease Control and Prevention, 2013; An et al., 2017). Briefly, genomic DNA was digested with 50 U of restriction enzyme XbaI (Roche Diagnostics, Indianapolis, IN, USA) for 2 h at 37°C. Electrophoresis was carried out using a 1% agarose gel in 0.5 X Tris-borate-EDTA buffer at 14°C in a CHEF Mapper XA System (BioRad Laboratories, Inc., Hercules, CA, USA) with the following conditions: 6 V/cm for 19 h with an initial switch time of 2.16 s and final switch time of 63.8 s. The analysis of the PFGE patterns was performed using BioNumerics software version 5 (Applied Maths, St.-Martens-Latern, Belgium). Similarities between PFGE patterns were determined based on the Dice similarity coefficient. The resulting similarities in the matrix were further processed by employing the unweighted-pair group method using average linkages to create a dendrogram that depicted the genetic relatedness between NTS isolates.

### Biofilm formation assay

Abilities of the NTS isolates to produce biofilms were assessed as previously described (O'Toole and Kolter, 1998). Briefly, overnight cultures of tested isolates were diluted 1:100 into LB medium, then dispensed into wells of 96-well polyvinyl chloride microtiter plates (Costar 2797, Corning, NY), followed by incubation at 37°C for 24 h. The cultures in 96-well plates were stained with 0.1% crystal violet for 10 min, followed by solubilization with 125 μl of 30% glacial acetic acid for 10 min. After this, the cultures were transferred to flat-bottom polystyrene microtiter plates (Greiner bio-one, Germany) and quantified by measuring absorbance at 550 nm (*A*550) in an Epoch Microplate Spectrometer (Biotek, Winooski, VT). A negative control well (e.g., containing growth medium only) was included in each PVC microtiter plate, and the absorbance value of this well was subtracted from the values of all test wells (Kadurugamuwa et al., 2003). Also, a positive control, using *S. enterica* subsp. *enterica* serovar Enteritidis ATCC 4931, was included in each 96-well plate.

### *fimA* and *iroN* full-length genes sequencing and non-synonymous single nucleotide polymorphism (nsSNP) analysis

Primers for the *fimA* and *iroN* genes, *fimA* (forward 5′–CAG GAT GCA GAG ATA ACT TTT CTG and reverse 5′–CTA GCG CCG CGC CTT TCC TTA TCA) and *iroN* (forward 5′–TGC CTT TTC CTT AAT TGA ATG ATA and reverse 5′–GCA GTG CAT TGC TGG ATA TCA GTC), were designed to flank ~ 120 bp up- and down-stream of the targeted genes, respectively. The amplicons were generated by Platinum *Taq* DNA polymerase (Thermo Fisher Scientific) and were prepared for DNA sequencing by the Prism BigDye Terminator cycle sequencing kit (Applied Biosystems, Foster City, CA, USA). The nucleotide sequences on both strands were determined using an ABI 3730x1 DNA analyzer (Genomics Center, University of Minnesota, Minneapolis, MN). Each strand was checked, then aligned with its complementary strand. A consensus DNA sequence was obtained using Clustal Omega (Larkin et al., 2007). The regions of homologous recombinations within the *fimA* and *iroN* genes were identified using a non-parametric recombination detection method, SiScan (Gibbs et al., 2000), as described earlier (Vidovic et al., 2011a). The annotated DNA sequences were exported into Molecular Evolutionary Genetics Analysis (MEGA) version 7 (Tamura et al., 2007) for the identification of nsSNPs. Phylogeny, based on nsSNPs, was inferred using the minimum evolution method (Rzhetsky and Nei, 1992). The neighbor-joining algorithm (Saitou and Nei, 1987) was used to generate the initial phylogenetic tree.

Nucleotide sequence translation was carried out using EMBOSS Transeq (Kearse et al., 2012) (the European Molecular Biology Laboratory—European Bioinformatics Institute; Hinxton, Cambridge, United Kingdom). Protein structure was predicted using Protter 2D prediction software (Omasits et al., 2014). Raptor X software was used to predict potential binding sites from an amino acid sequence input based on prevalent predetermined binding motifs that correlate to a bank of small molecules (Källberg et al., 2012).

### Statistical analysis

To determine statistically significant differences in the proportion of genes and important nsSNPs between avian, bovine, and porcine NTS isolates, we used the Agresti-Coull method together with Fisher's exact test (Agresti, 2002). To test for an association between host origin and the ability of NTS isolates to form biofilm, we used a chi-squared test, both overall and pairwise between hosts groups.

### Nucleotide sequence accession numbers

Nucleotide sequences were deposited in GenBank. Accession numbers for the DNA sequences of the *fimA* and *iroN* genes ranged from KY367267 to KY367280.

## Results

### Virulence gene profiles of the NTS isolates

**Figure 1:**
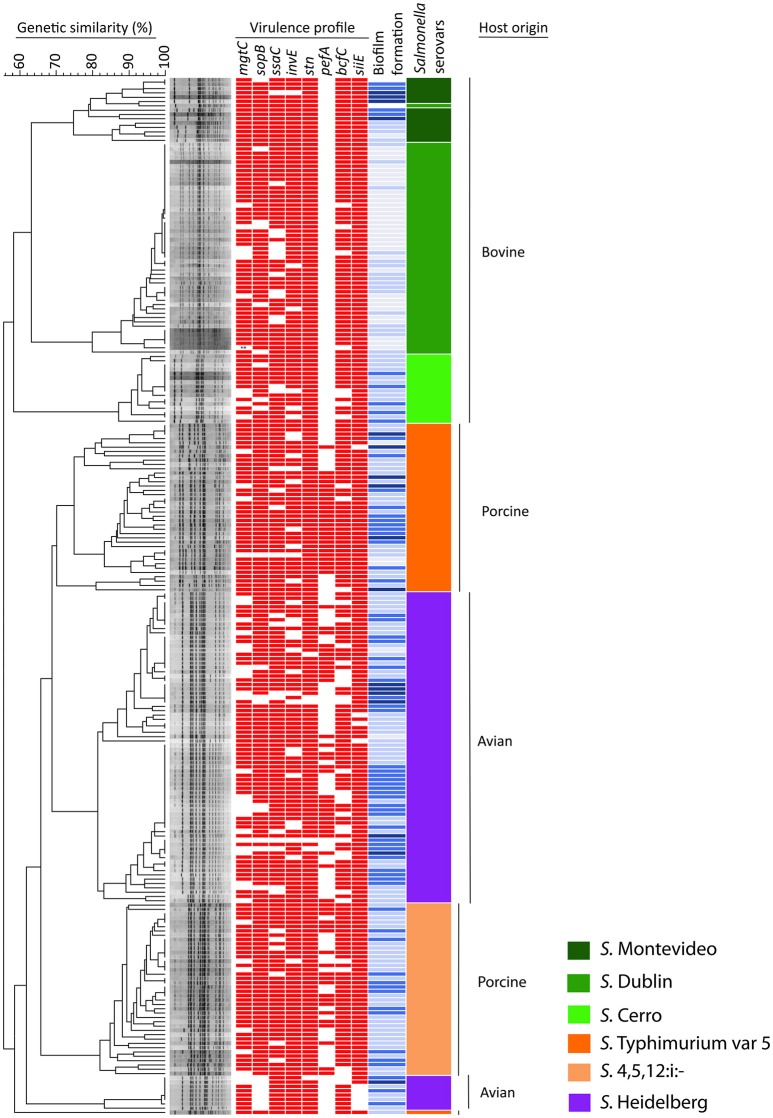
Dendrogram, virulence profiles and biofilm production in 240 NTS isolates of avian, bovine and porcine origin. The dendrogram is based on PFGE analysis of 80 clinical isolates of bovine origin (serovars Montevideo, Dublin, and Cerro), 80 clinical isolates of porcine origin (serovars Typhimurium var 5–and 4,5,12:i:–) and 80 avian-associated isolates (serovar Heidelberg). The virulence profile of each NTS strain is portrayed by a color-coded pattern (red indicates presence and no color indicates absence of the virulence factor). The ability of each NTS strain to form biofilm is presented by different shades of blue color (dark blue = high biofilm producers, blue = moderate biofilm producers, topaz sky blue = low biofilm producers, and light blue = no biofilm produced). The virulence profile and ability to produce biofilm in each NTS strain was aligned with its position in the dendrogram. The serovar identity of each isolate is presented by one of six different colors. Vertical bars on the far right indicate the different host origins represented in this NTS collection.

The occurrence of the eight virulence-associated genes, *pefA, siiE, stn, sopB, bcfC, mgtC, ssaC*, and *invE*, among 240 NTS isolates is shown in Table 3, Figure 1. The *stn* gene, which encodes enterotoxin, had the highest frequency of occurrence in all three groups of NTS isolates. All S. Dublin, S. Montevideo, S. Cerro, and S. 4,5,12,:i:- isolates, almost all S. Typhimurium var 5- isolates and a majority of *S*. Heidelberg isolates were positive for the *siiE* gene that encodes giant non-fimbrial adhesion protein. Another highly prevalent (>80%) virulence factor was the effector gene *sopB*, required for host invasion. High frequencies of occurrence (>80%) were also observed for the *ssaC* gene (i.e., secretion system apparatus outer membrane protein C) and the *invE* gene (i.e., invasion protein). The *mgtC* gene (i.e., magnesium transporter) was found in most bovine (95%), porcine (87.7%), and avian isolates (67.5%); this difference was significant (*P* = 0.00). The virulence-associated gene, fimbrial usher *bcfC*, had a high frequency of occurrence in *S*. Montevideo (100%), *S*. Dublin (92%), *S*. Typhimurium var 5- (90%), *S*. 4,5,12,:i:- (87.5%), but relatively lower prevalence in *S*. Cerro (75%) and *S*. Heidelberg (70%). Also, this difference was statistically significant (*P* = 0.00). Occurrence of the *pefA* gene (e.g., plasmid-encoded major fimbrial subunit) showed the most significant difference (*P* < 0.00) comparing *S*. Typhimurium var 5- (64.1%), *S*. 4,5,12,:i:- (48.8%), and *S*. Heidelberg (38.7%) to the bovine isolates *S*. Dublin. *S*. Montevideo and *S*. Cerro in which the gene was absent.

**Table 3:** Distribution of virulence-associated genes within the collection of NTS isolates of avian, bovine and porcine origins.

**Virulence genes**	**No. (%) of isolates positive for virulence-associated genes**				
	**Total (*n* = 240)**	**Avian (*n* = 80)**	**Bovine (*n* = 80)**	**Porcine (*n* = 80)**	***P*-values**
*pefA*	76 (31.6)	31 (38.7)	0	45 (56.2)	0.00
*siiE*	230 (95.8)	71 (88.7)	80 (100)	79 (98.7)	0.00
*stn*	234 (97.5)	74 (92.5)	80 (100)	80 (100)	0.01
*sopB*	214 (89.1)	64 (80)	71 (88.7)	79 (98.7)	0.00
*bcfC*	194 (80.8)	56 (70%)	72 (90)	73 (91.2)	0.00
*mgtC*	200 (83.3)	54 (67.5)	69 (86.2)	70 (87.5)	0.00
*ssaC*	206 (85.8)	71 (88.7)	65 (81.2)	70 (87.5)	0.19
*invE*	208 (86.7)	64 (80)	74 (92.5)	70 (87.5)	0.07

### Ability of the NTS isolates to form biofilms

**Figure 2:**
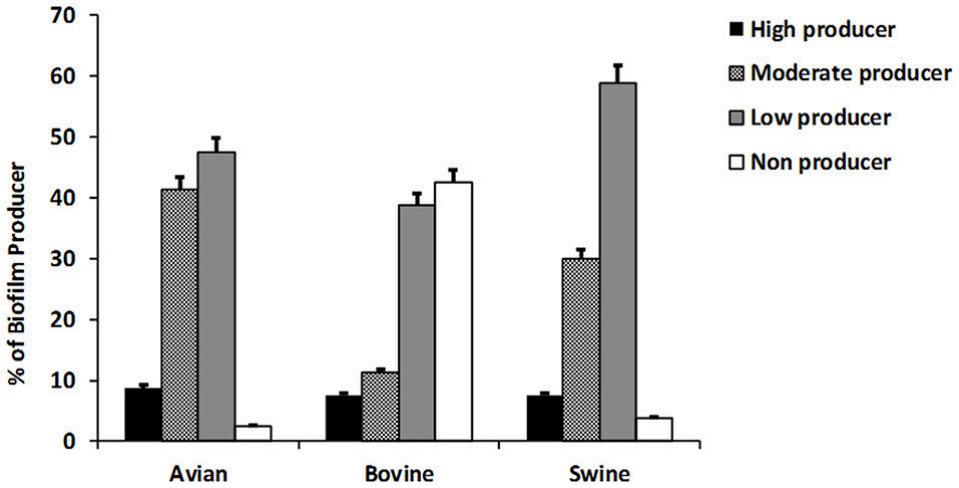
The ability of NTS isolates of different host origins to form biofilms. The biofilm formation of all NTS isolates tested in this study was compared to that of *Salmonella enterica* subsp. Enterica serovar Enteritidis ATCC® 4931 (OD = 0.090333). The values of NTS isolates that correlated to the absorbance of ≤0.068 were considered as weak producers, 0.069–0.136 as moderate producers and ≥0.137 as strong producers. The results are representative of three independent experiments carried out in triplicate.

The results of this assay are shown in Figure 2 collectively, for each host-associated group (e.g., avian, bovine, and porcine) and each individual NTS strain in Figure 1. Populations of NTS isolates from the three hosts contained a similar percentage (7.5–8.75%) of the high biofilm-producing isolates. Among the group of moderate biofilm-producing isolates, *S*. Heidelberg were highest (41.2%), followed by S. Cerro (31.2%), *S*. Typhimurium var 5- (30%), S. 4,5,12,:i:- (30.0%), *S*. Montevideo (28.5%), and *S*. Dublin (0%) (*P* < 0.05). The low biofilm producers were similarly distributed among NTS of porcine (58.7%), avian (47.5%), and bovine (38.7%) origin. NTS isolates that did not produce biofilms represented a smallest fraction among *S*. Typhimurium var 5- (7.5%), *S*. Montevideo (7.1%), *S*. Heidelberg (2.5%), *S*. Cerro (0%), and *S*. 4,5,12,:i:- (0%), but were most frequent among *S*. Dublin (66%) (*P* < 0.005; Figure 1).

### Overall genetic relatedness of the NTS isolates

The 240 NTS isolates were resolved by PFGE into 137 pulsotypes, which were further grouped into 10 clusters and 1 outlier (Figure 1). The NTS isolates of bovine origin were resolved into 41 pulsotypes, grouped into three clusters and placed at the apical part of the dendrogram. Each of three clusters was exclusively comprised of isolates that belonged to a single serovar (i.e., except the top cluster which possesses 14 isolates of *S*. Montevideo and one isolate of *S*. Dublin). The top cluster was comprised of Montevideo isolates with 75% genetic similarity, the second top cluster contained Dublin isolates with 76% genetic similarity and the third, most homogeneous cluster was comprised of Cerro isolates with 83% genetic similarity. The NTS isolates of porcine origin were split into three monophyletic clusters, located at the central part of the dendrogram, and another cluster positioned at the basal part of the dendrogram. The three monophyletic clusters were comprised of Typhimurium var 5- isolates with 68% genetic similarity. Besides these three monophyletic clusters, one genetically distant isolate of S. Thyphimurium var 5- formed an outlier and shared 58% of genetic similarity with the rest of Salmonella isolates. The cluster located at the basal part of the dendrogram contained isolates of 4,5,12:i:- serovar with 78% genetic similarity. *Salmonella* Heidelberg isolates were resolved into two large, genetically related clusters and third, much smaller cluster. Two genetically related clusters were placed at the central part of the dendrogram, while the smaller cluster was positioned at the bottom of the dendrogram. Heidelberg shared 62% genetic similarity among themselves, which made this group of *Salmonella* the most heterogeneous among the entire collection of NTS isolates although comprised of one serotype. Interestingly, *S*. Heidelberg contained multiple pulsotypes that grouped different isolates within the same pulsotype, indicating that *S*. Heidelberg may have several clonal groups (Figure 1). Some of these clonal groups are genetically related (e.g., pulsotypes within two large clusters), whereas others are genetically distant (e.g., pulsotypes between the small and two large clusters) (Figure 1).

### Identification of nsSNPs and determination of their impact on the fima adhesin and the iron receptor

**Figure 3:**
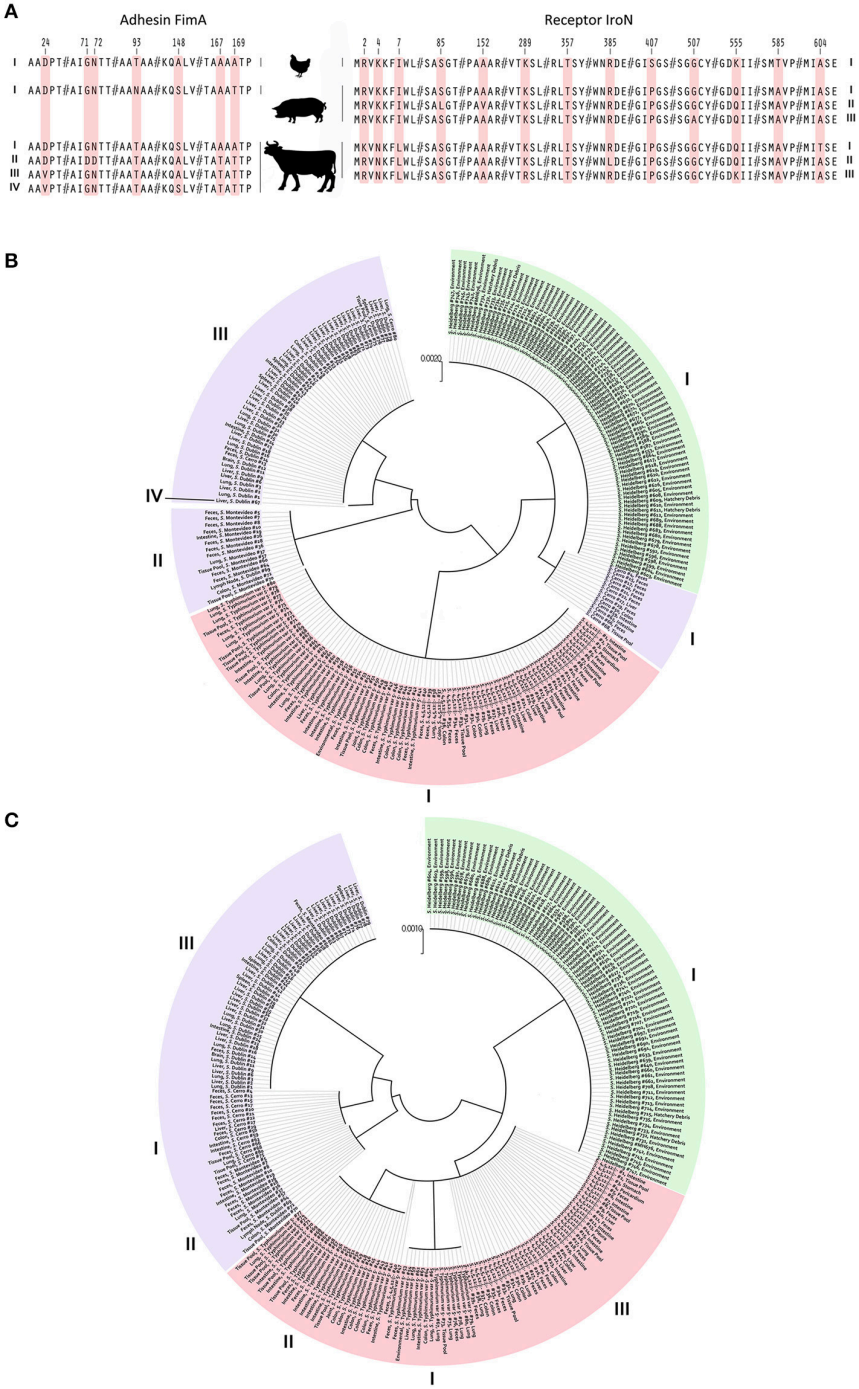
Identification of nsSNPs and their influence on adhesin FimA and receptor IroN of NTS isolates from avian, bovine and porcine hosts. **(A)** Image portrays amino acid substitutions in different FimA and IroN haplotypes of NTS isolates obtained from avian, porcine and bovine hosts. Each amino acid substitution is coded by light red color and numbers above indicate the positions of point mutations within the FimA and IroN proteins. On the far left and far right are Roman numerals indicating FimA and IroN haplotype numbers, respectively. **(B)** Phylogeny of the FimA adhesin. Each of 240 NTS isolates is marked by its identification, name of serovar, and site of isolation. NTS of avian origin are colored green, porcine light red, and bovine light purple. Roman numerals positioned around the phylogenetic tree indicate the identity of different haplotypes. **(C)** Phylogeny of the IroN receptor. An identical approach was used to mark different NTS groups and their IroN haplotypes as was done with FimA.

To avoid any effect of homoplasy on the SNP analysis, all DNA sequences were tested for regions of homologous recombination. A total of six alleles, among 240 NTS isolates, were identified for the *fimA* gene. The overall average pairwise distance for the *fimA* gene, an estimate of evolutionary divergence between sequences was 0.0082 among *S*. Dublin, *S*. Montevideo, and *S*. Cerro. Evolutionary divergence in the same gene among *S*. Heidelberg, *S*. Typhimurium var 5- and *S*. 4,5,12,:I:- did not exist. No SNP was found among the populations of *S*. Heidelberg, *S*. Typhimurium var 5- and *S*. 4,5,12,:I: isolates. Fourteen SNPs were identified among *S*. Dublin, *S*. Montevideo and *S*. Cerro isolates, resulting in a SNP density of 40 (one SNP per 40 bp). Out of these 14 SNPs, six were nsSNPs, further generating four haplotypes of protein FimA among the bovine isolates. These four haplotypes were compared with *S*. Heidelberg, *S*. Typhimurium var 5- and *S*. 4,5,12,:i:- haplotypes, and seven amino acid substitutions were identified at residues 24, 71, 72, 93, 148, 167, and 169 of the FimA protein (Figure 3A). An amino acid substitution at the 93 position of the FimA protein was unique to NTS isolates of porcine origin. Another six amino acid substitutions were shared between NTS isolates from all six *Salmonella* serovars (Figure 3A).

Protein-based phylogeny of the FimA adhesin showed that each *S*. Heidelberg and *S*. Typhimurium var 5- as well as *S*. 4,5,12,:i:- together was grouped into a single distinct cluster, whereas the bovine isolates were grouped into three major clusters and a single outlier (Figure 3B). The FimA adhesin of the great majority (*n* = 67, 84%) of bovine isolates was clustered into a clade, genetically most diverged from other NTS isolates. Most of the *S*. Cerro isolates were clustered together with *S*. Heidelberg isolates into a clade (Figure 3B). In contrast to the bovine isolates, NTS isolates of avian and porcine origins showed profound homogeneity of FimA adhesin (Figure 3B).

The full-length sequencing of the *iroN* gene (e.g., 2,175 nt) revealed the existence of eight alleles among the population of 240 NTS isolates. Each population of bovine and porcine isolates was composed of three unique alleles, while the population of avian isolates contained two alleles of the *iroN* gene. The overall average pairwise distance of the *iroN* gene was 0.00817, 0.00046, and 0.00001 for the bovine, porcine and avian isolates, respectively, indicating that any two bovine isolates would diverge, on average, ~0.82%, porcine 0.04% and avian 0.001%. Among the isolates of bovine origin, 47 SNPs were identified within the *iroN* gene, while avian and porcine *iroN* sequences contained one and three SNPs, respectively. *Salmonella* Dublin, *S*. Montevideo, and *S*. Cerro isolates had the highest SNP density of 46, followed by *S*. Typhimurium var 5- and *S*. 4,5,12,:I:- isolates of 725; *S*. Heidelberg isolates had the lowest SNP density of 2,175. Out of 47 SNPs identified in *iroN* among *S*. Dublin, *S*. Montevideo and *S*. Cerro isolates, six were nsSNPs. All three *iroN* SNPs among *S*. Typhimurium var 5- and *S*. 4,5,12,:i:- isolates were nsSNPs, and no nsSNPs were detected among *S*. Heidelberg isolates. In total, a single IroN haplotype was found among *S*. Heidelberg isolates and three IroN haplotypes each were found among *S*. Dublin, *S*. Montevideo, *S*. Cerro, and *S*. Typhimurium var 5- as well as *S*. 4,5,12,:i:- isolates. When these seven haplotypes were compared, 13 amino acid substitutions were identified at residues 2, 4, 7, 85, 152, 289, 357, 385, 407, 507, 555, 585, and 604 of the IroN protein (Figure 3A). Amino acid substitutions at positions 4 and 7 were unique in *S*. Dublin, *S*. Montevideo, *S*. Cerro isolates and substitutions at positions 407 and 585 were unique in *S*. Heidelberg isolates (Figure 3A).

Phylogeny of the IroN receptor showed the existence of three clades, each representing a group of NTS isolates of distinct host origin (Figure 3C), indicating a greater interdependence between the phylogeny and the host origin of NTS isolates compared to that of FimA adhesin.

A summary of characteristics for each haplotype of FimA and IroN, including: solvent accessibility, type of ligand, predicted ligand-protein binding sites and *P* value of predicted model, is presented in Table 4. There is a major division among the haplotypes of FimA adhesin based on their predicted binding sites. FimA of *S*. Heidelberg isolates (*P* = 6.27e-07) and *S*. Cerro isolates (*P* = 1.10e-07) preferably bind to N-acetyl-D-glucosamine, whereas FimA adhesin of *S*. Typhimurium var 5−*and S*. 4,5,12,:i:- isolates (*P* = 4.24e-07) and *S*. Dublin (*P* = 5.19e-07) and *S*. Montevideo (*P* = 1.22e-07) bind preferentially to a different ligand, chloramphenicol. Among the first group, the solvent accessibility analysis showed that the FimA protein of *S*. Heidelberg isolates was buried by 23% compared to 31% in the haplotype of *S*. Cerro, indicating better solvent accessibility for the FimA of *S*. Heidelberg isolates. Among the second group, the FimA of *S*. Montevideo haplotype was most buried (34%), compared to other two FimA haplotypes, Typhimurium var 5-, 4,5,12,:i:- and Dublin (22 and 23%, respectively). All haplotypes of the IroN receptor had profound affinity to Fe^3+^ ion (Table 4). The solvent accessibility analysis showed that only the IroN of *S*. Dublin haplotype had lower solvent accessibility, 35% of protein being buried, compared to 24 and 23% for other IroN haplotypes (Table 4).

**Table 4:** Characteristics of avian, bovine and porcine FimA and IroN haplotypes.

**Haplotype name**	**Solvent accessibility (%)**			**Ligand**	**Predicted binding sites**	***P*-values**
	**Exposed**	**Medium**	**Buried**			
FimA_Avian	29	47	23	N-acetyl-D-glucosamine	A145, Q147, A148, L149, V150, T153, N154, T155, L156	6.27e-07
FimA_Porcine	29	48	22	Chloram-phenicol	A75, Q76, V77, P78, R157, T159, A160, R161	4.24e-07
FimA_Bovine-1	27	41	31	N-acetyl-D-glucosamine	A145, Q147, A148, L149, V150, T153, N154, T155, L156	1.10e-07
FimA_Bovine-2	29	47	23	Chloram-phenicol	A75, Q76, V77, P78, T159, A160, R161	5.19e-07
FimA_Bovine-3	27	38	34	Chloram-phenicol	A75, Q76, V77, P78, R157, T159, A160, R161	1.22e-07
IroN_Avian	40	34	24	Fe^3+^	N82, T87, R88, V119, R120, Y121, S122, W123, R124, G125, E126, R127, D128, R332, E335	1.29e-32
IroN_Porcine-1	39	35	24	Fe^3+^	N82, T87, R88, V119, R120, Y121, S122, W123, R124, G125, E126, R127, D128, R332, E335	1.08e-32
IroN_Porcine-2	40	35	24	Fe^3+^	N82, T87, R88, V119, R120, Y121, S122, W123, R124, E126, R127, D128, Q280, R332, E335	4.28e-33
IroN_Porcine-3	40	35	24	Fe^3+^	N82, T87, R88, E102, V119, R120, Y121, S122, W123, R124, E126, R127, D128, Q280, E335	1.51e-32
IroN_Bovine-1	39	35	24	Fe^3+^	N82, T87, R88, E102, V119, R120, Y121, S122, W123, R124, E126, R127, D128, R332, E335	1.05e-32
IroN_Bovine-2	40	35	23	Fe^3+^	N82, T87, R88, E102, V119, R120, Y121, S122, W123, R124, E126, R127, D128, T129, E335	1.05e-32
IroN_Bovine-3	40	23	35	Fe^3+^	N82 T87 R88 V119 R120 Y121 S122 W123 R124 E126 R127 D128 T129 Q280 E335	3.36e-33

## Discussion

In this host-NTS population based study, we examined the role of nsSNPs in the process of adaptation of NTS to their hosts and revealed genetic as well as virulence signatures of NTS groups based on their serovar identity. Using a well-defined population of 240 NTS isolates in combination with full gene sequencing of two important antigens, adhesin *fimA* and salmochelin receptor *iroN*, we discovered a key nsSNP in the *fimA* that profoundly shifts in ligand preference of the adhesin FimA. Multiple alignments of all FimA haplotypes clearly showed that amino acid substitution at position 169, threonine (T) to alanine (A), produced a significant shift in ligand affinity of adhesin FimA from chloramphenicol to N-acetyl-D-glucosamine, a glucose derivative involved in cell wall biogenesis of many prokaryotic and eukaryotic organisms. This nsSNP occurred exclusively in the FimA haplotype of *S*. Heidelberg and *S*. Cerro, indicating a further phylogenetic segregation of these two FimA haplotypes from other adhesin haplotypes, forming a single clade. It is noteworthy to mention that although Heidelberg and Cerro FimA haplotypes showed phylogenetic similarity and identical ligand preference, these two haplotypes differ in solvent accessibility. The Heidelberg FimA haplotype is less buried and more exposed to solvents than Cerro FimA haplotype, which may indicate the host specificity of these two FimA haplotypes. To gain an insight into the influence of the three different hosts (i.e., immune selective pressure) on these two antigen genes, we performed the PFGE analysis and compared it to the phylogeny of the FimA and IroN proteins. The PFGE analysis provides an overall genetic relatedness of bacterial isolates based on their entire genomes, which is mostly not affected by the host but rather by a general genome make up of a bacterial isolate. In contrast to the PFGE analysis, the phylogeny of the FimA adhesion and the IroN receptor, provides a direct insight into the influence of the host's immune selective pressure and microbiome on these two outer membrane proteins. By comparing the phylogeny of the FimA adhesin with the PFGE dendrogram, we observed a profound homogeneity of the FimA adhesin of *S*. Heidelberg, *S*. Typhimurium var 5- and *S*. 4,5,12,:i:-. The PFGE analysis clustered *S*. Typhimurium var 5- and *S*. 4,5,12,:i:- isolates into two large clusters, separated by a large cluster of *S*. Heidelberg, whereas the FimA adhesin of the same NTS isolates was clustered as a single FimA allele, indicating a strong influence of the host on this adhesin. Similarly, the population of *S*. Heidelberg isolates was resolved into two clusters by the PFGE analysis, with genetic similarity of 62%, indicating an existence of the genetically very heterogeneous population. In contrast to the PFGE analysis, the phylogeny of the FimA protein of *S*. Heidelberg resulted in a distinct homogenous allele, again indicating a substantial influence of the particular host's immune pressure on this outer membrane protein. Interestingly, a single FimA allele of *S*. Cerro showed a closer relation to the FimA allele of *S*. Heidelberg, which may suggest that this small group of bovine isolates may equally affect the avian host. Another possible explanation is that this group of bovine isolates has been exposed to the bovine host for a short period.

It has been shown that nsSNPs in epitopes of certain antigens can lead to profound changes in biological fitness of different microbial pathogens, resulting in the emergence of highly virulent isolates (Kim et al., 2003; Choi et al., 2006; Fujimoto et al., 2008; Vidovic et al., 2011b) or determination of the pathogen's host tropism (Yue et al., 2015). For instance, Choi et al. (2006), examining genetic heterogeneity in the hexon gene of human adenovirus type 3 (Ad3) over a 9-year period in Korea, discovered novel, emerging Ad3a16 and Ad3a18 genotypes during an Ad3 outbreak of childhood pneumonia in Korea. Newly emerged Ad3a16 and Ad3a18 genotypes had three amino acid substitutions in loop 2 of the hexon gene. These three nsSNPs were associated with greater hydrophobicity of this protein, resulting in the replacement of the previously predominant Ad3 genotypes by newly emerged Ad3a16 and Ad3a18, apparently due greater fitness. Most recently, Yue and colleagues (Yue et al., 2015) found that a single amino acid substitution in the binding pocket of FimH, the type 1 fimbrial adhesin, can dramatically change ligand affinity of this adhesin and result in determination of the NTS host tropism toward mammalian or avian host receptors. It has been shown that the presence of isoleucine instead of threonine at position 78 of the FimH in Gallinarum and Pullorum serovars affects mannose-inhibitable binding (Kisiela et al., 2005), and may determine the avian host specificity of these two NTS serovars (Guo et al., 2009; Kisiela et al., 2012; Kuzminska-Bajor et al., 2012).

We identified numerous nsSNPs in the *iroN* gene that had no influence on Fe^3+^ affinity or solvent accessibility among the IroN haplotypes of NTS groups from avian, bovine and porcine hosts. All IroN haplotypes showed an extremely high affinity for Fe^3+^ and great solvent accessibility, which can be explained by the functional constraint of this receptor. In other words, the primary role of IroN is Fe^3+^ acquisition, regardless of NTS host origin. It is important to mention that despite a relatively high heterogeneity of the IroN receptor, all IroN haplotypes showed a strong correlation between their phylogeny and the host origin of NTS isolates. Each IroN haplotype was clustered together with other haplotypes from the same host-origin group (Figure 3C). Comparing the IroN phylogeny to that of the FimA, it is evident that the host influence is even greater on the IroN receptor of NTS isolates, as numerous IroN haplotypes were clustered exactly according to their host origin. This strong connection between phylogeny of IroN and the host origin of NTS isolates can be explained by the location and function of this protein. IroN, an outer membrane siderophore receptor unique for *S. enterica* subspecies I, II, IIIa, IIIb, IV, and VI (Baumler et al., 1998), represent an ideal target for the host immune system (Fernandez-Beros et al., 1989; Heithoff et al., 1997). Besides its primary role in Fe^3+^ acquisition, IroN is responsible for acquiring metabolites secreted by other bacteria, such as myxochelin A (Corbin and Bulen, 1969), 2-N,6-N-bis(2,3-dihydroxybenzoyl)-L-lysine and 2-N,6-N-bias(2,3-dihydroxybenzoyl)-L-lysine amide (Kunze et al., 1989), providing a growth advantage specifically to *S. enterica* subspecies I, II, IIIa, IIIb, IV, and VI in the gut microbiota. A stronger influence of the host on the IroN receptor compared to the FimA adhesin can be explained by the fact that the IroN receptor is under selective pressure of both, the host immune system and the host's gut microbiota, whereas FimA is under pressure of the host's immune system only. Taken together, nsSNPs identified among the IroN haplotypes of different NTS host-origin groups may represent an evolutionary response to both the immune selective pressure of the hosts and their unique microbiotas, as IroN is involved in acquisition of secondary metabolites produced by the host microbiota, which can explain the association between the IroN phylogeny and the host origin of NTS.

Evaluation of the genetic relatedness among 240 NTS isolates of *S*. Heidelberg, *S*. Dublin, *S*. Montevideo, *S*. Cerro *S*. Typhimurium var 5- and *S*. 4,5,12,:I:- by PFGE analysis revealed that the isolates of *S*. Heidelberg were the most heterogeneous group, composed by multiple closely and distantly related clones. In a sharp contrast to their overall genetic heterogeneity, *S*. Heidelberg isolates showed a remarkable genetic homogeneity of their two antigens, adhesin FimA and receptor IroN, with only one single allele in each protein. The similar agreement between the overall genetic makeup and the genetic diversity of FimA and IroN was observed for *S*. Typhimurium var 5- and S. 4,5,12,:i:- isolates. The data presented here show that the nsSNPs in two outer membrane proteins, FimA and IroN, are based on serovar identity, driven by host specificity which most likely play roles in the process of NTS adaptation to their specific hosts.

Both the Agresti-Coull method and Fisher's exact test strongly suggested that among all of the tested virulence factors, the prevalence of *pefA* was the most segregative characteristic of the tested NTS population. Although the bovine NTS isolates were composed of three serovars, Dublin, Montevideo and Cerro, not a single isolate was found to carry *pefA*, a gene encoding a major fimbrial antigen subunit (Woodward et al., 1996) whereas the same virulence factor was detected in NTS isolates from avian and porcine hosts. This virulence factor most likely has an impact on the ability of NTS isolates to form biofilms, an important phenotype in overall NTS pathogenicity, as the NTS isolates of bovine origin had a reduced ability to form biofilm compared to that of the other two NTS hosts. This significant difference in the presence/absence of the *pefA* virulence gene among the collection of NTS isolates was phenotypically confirmed. NTS isolates that did not produce biofilms were significantly associated with NTS isolates of the bovine origin.

In summary, the present study has determined a key amino acid substitution, A169, in FimA that shifted the affinity of this adhesin toward N-acetyl-D-glucosamine, a glucose derivative in the cell wall matrix of prokaryotic and eukaryotic organisms. This mutation was significantly associated with *S*. Heidelberg and *S*. Cerro, suggesting that A169 may play a role in seovar and host specificity of NTS. The results also indicate that there is a strong positive association between the phylogeny of the IroN receptor and different host origins of NTS isolates, indicating an influence of immune selection pressures imposed by a specific host on this unique receptor of *S. enterica*. Through screening some of the most important virulence factors among a large collection of NTS isolates, we found that *pefA* was the most significant determinant segregating NTS isolates from different hosts. This virulence factor, involved in NTS adherence and biofilm formation, was completely absent from NTS isolates of the bovine origin, which most likely resulted in the significant percentage of the population of bovine isolates being unable to produce biofilm.

## Author contributions

SV and CC conceived the study. SA, SH, RA, JM, CF, and RN carried out experiments. DL and KO provided non-typhoidal *Salmonella* (NTS) isolates. SV, SA, RA, SH, and DB analyzed the data. SV and JA facilitated the collaborative project. SV drafted the manuscript. All authors read and approved the final version of the manuscript.

### Conflict of interest statement

The authors declare that the research was conducted in the absence of any commercial or financial relationships that could be construed as a potential conflict of interest.
